# A Precisely Controlled Long-Acting Immunosuppression Platform Enables Prolonged Survival of Vascularized Composite Allografts

**DOI:** 10.1101/2025.11.07.687240

**Published:** 2025-11-10

**Authors:** Sohyung Lee, Fatih Zor, Yalcin Kulahci, John Joseph, Huseyin Karagoz, Shumaim Barooj, Gunjan Malik, Dongsung Park, James N Luo, Lingyun Zhu, Ally Wang, Krisco Chung, Nancy Li, Purna Shah, Eli Agus, Gopinathan Janarthanan, Sanjairaj Vijayavenkataraman, Helna Mary Baby, Olivia Snapper, Xinyang Chen, Kai Slaughter, Mickael Dang, Ethan Sam, Amogh Gorantla, Wensheng Zhang, Jeffrey M. Karp, Nitin Joshi, Vijay Gorantla

**Affiliations:** 1 Harvard Medical School, Boston, MA, USA; 2 Center for Accelerated Medical Innovation, Department of Anesthesiology, Perioperative and Pain Medicine, Brigham and Women’s Hospital, Boston, MA 02115, USA; 3 Center for Nanomedicine, Department of Anesthesiology, Perioperative and Pain Medicine, Brigham and Women’s Hospital, Boston, MA, USA; 4 Departments of Surgery, Ophthalmology and Bioengineering, Wake Forest University, USA.; 5 Department of Plastic and Reconstructive Surgery, Gulhane Military Medical Academy, Haydarpasa Training Hospital, Istanbul, Turkey.; 6 Department of Electrical Engineering and Computer Sciences, University of California Berkeley, Berkeley, California 94720, USA.; 7 Department of Surgery, Brigham and Women’s Hospital, Boston, MA 02115, USA; 8 The Vijay Lab, Division of Engineering, New York University Abu Dhabi, Abu Dhabi, United Arab Emirates.; 9 Department of Biomedical Engineering, Tandon School of Engineering, New York University, Brooklyn, NY 11201, USA.; 10 Department of Mechanical and Aerospace Engineering, Tandon School of Engineering, New York University, Brooklyn, NY 11201, USA.; 11 McGowan Institute for Regenerative Medicine, University of Pittsburgh, USA.; 12 Wilford Hall Ambulatory Surgical Center, 59th Medical Wing Office of Science and Technology, Joint Base San Antonio, USA.; 13 Harvard–Massachusetts Institute of Technology Division of Health Sciences and Technology, Massachusetts Institute of Technology, Cambridge, MA 02139, USA.; 14 Broad Institute, Cambridge, MA 02142, USA.; 15 Harvard Stem Cell Institute, Cambridge, MA 02138, USA

## Abstract

Vascularized composite allotransplantation (VCA) restores complex tissue defects but demands lifelong systemic immunosuppression. Oral tacrolimus (TAC) is limited by a narrow therapeutic window, pharmacokinetic variability, adherence challenges, and toxicity. Local delivery could mitigate these issues, yet clinical translation has been hindered by burst release, short duration, and the inability to co-deliver agents.

We developed PRECISE (Programmable, REtrievable, Controlled ImmunoSuppression Encapsulator), an injectable, in-situ–forming PLGA depot that achieves long-acting, tightly controlled TAC release via structure-guided co-formulation with drug-binding agents (DBAs). GRAS small molecules (e.g., EGCG, maltotriose) identified by in silico docking and in vitro screening suppressed burst and modulated solvent efflux. Notably, rapamycin (RAPA) served dually as an mTOR inhibitor and a TAC-binding excipient, enabling synchronized dual-agent delivery.

PRECISE eliminated burst in vitro and produced coordinated TAC+RAPA release with clinically compatible injectability. In rats, monthly intragraft dosing maintained systemic TAC/RAPA within the therapeutic window (~5–10 ng/mL) for >300 days, prolonged hindlimb allograft survival, expanded Tregs, and induced donor-specific hyporesponsiveness. Surgical retrieval of the depot triggered rapid TAC decline, demonstrating reversibility. In a stringent, fully MHC-mismatched porcine VCA model, PRECISE maintained on-target drug levels and extended graft survival beyond 90 days with minimal rejection and preserved vascular integrity.

PRECISE is, to our knowledge, the first retrievable, injectable platform to deliver long-acting, dual-agent immunosuppression with controlled kinetics, rapid attainment of therapeutic steady state, and sustained graft protection. Its modular, structure-guided design enables clinical translation across VCA and solid-organ transplantation, delivering precise, durable, and safer immunosuppression.

## Introduction

Vascularized composite allotransplantation (VCA) restores complex defects comprising multiple functional tissues such as skin, muscle, bone, and nerves that are critical for mobility and sensation, offering substantial improvement in patient quality of life. Despite surgical success, VCA recipients remain highly susceptible to both acute and chronic rejection due to the immunologic complexity of multi-tissue grafts, with varying antigenicity and vascularization profiles, which complicate immune regulation and heighten the risk of rejection (1). Immunosuppressive therapy is therefore essential, with oral tacrolimus (TAC), a calcineurin inhibitor, serving as the mainstay of most VCA protocols.(2, 3) However, TAC has a narrow therapeutic window (5–15 ng/mL),(4, 5) and post-dose peak concentrations can lead to periods of over-immunosuppression, increasing the risk of nephrotoxicity, malignancy, and opportunistic infection (5). Additionally, oral TAC demonstrates substantial interpatient pharmacokinetic variability due to differences in gastrointestinal absorption and first-pass metabolism, resulting in unpredictable drug exposure and alternating periods of under- or over- immunosuppression (6). Finally, suboptimal compliance with once- or twice-daily dosing can lead to missed doses and dangerous lapses in immunosuppression, further elevating the risk of graft rejection (7). These limitations underscore the necessity for a strategy that can maintain TAC concentrations precisely and stably within the therapeutic window over an extended duration, thereby minimizing the risks of both toxicity and rejection while enhancing patient compliance.

Sustained, long-acting drug delivery strategies represent a promising solution to overcome the limitations of oral immunosuppressive therapies. Formulations administered subcutaneously or intramuscularly can provide prolonged, controlled drug release while bypassing gastrointestinal absorption and first-pass metabolism (8, 9). In the setting of VCA, these systems can also be administered directly at or near the graft site, which allows them to achieve high local TAC concentrations while simultaneously maintaining therapeutic concentrations systemically. Graft-embedded or peri-graft controlled-release systems are particularly attractive because they could accomplish both local immunoregulation and systemic immunosuppression (10).

Several approaches have been explored for sustained graft-embedded immunosuppression in VCA (2, 11, 12). Although these systems have shown preclinical promise in improving graft survival and reducing dosing frequency, they often exhibit pronounced systemic burst release of TAC and fail to achieve steady-state drug levels rapidly, resulting in prolonged supratherapeutic exposure and increased risk of adverse effects (13, 14). Moreover, although combination regimens with complementary mechanisms of immunosuppression have demonstrated superior efficacy over monotherapy (15), sustained delivery platforms for immunosuppressive therapies remain predominantly limited to single-drug formulations.

**Figure 1.**
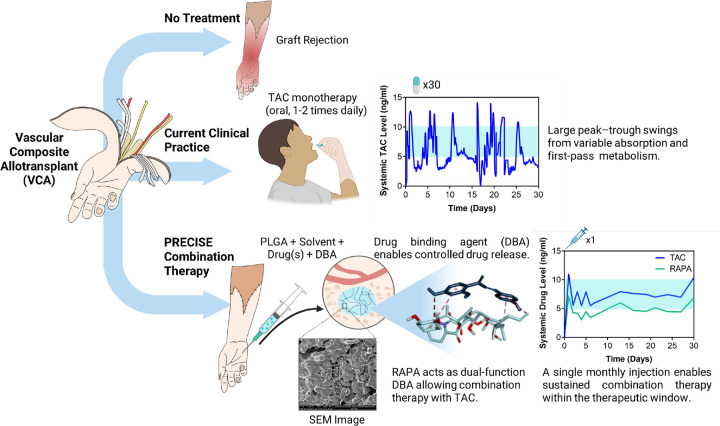
PRECISE—PLGA-based, in situ–forming depot for localized transplant immunosuppression. The current standard of care in VCA relies on daily oral TAC, which exhibits fluctuating systemic levels and risks toxicity or under-immunosuppression. In contrast, a single peri-/intra-graft injection of the PRECISE formulation solidifies in situ to create a localized drug reservoir (as shown in SEM image) that sustains TAC (and RAPA) within the therapeutic window for months. DBAs, selected via structure-guided molecular interaction screening, attenuate burst release and prolong TAC exposure. RAPA, when co-encapsulated, can function both as an immunosuppressive agent and as a DBA to enable combination therapy with coordinated, sustained release.

We introduce PRECISE – Programmable, REtrievable, Controlled ImmunoSuppression Encapsulator, an injectable, graft-embedded formulation engineered to provide long-acting release of one or more immunosuppressive agents while maintaining systemic drug levels precisely within the therapeutic window. PRECISE comprises an *in-situ* forming matrix of poly(lactic-co-glycolic acid (PLGA) dissolved in a biocompatible solvent and co-formulated with drug-binding agents (DBAs) that finely regulate the release kinetics of the encapsulated TAC. Upon injection, PRECISE undergoes phase inversion, rapidly forming a porous, solid depot at the graft site through solvent efflux, enabling controlled and sustained drug release (Figure 1).

DBAs were identified through a structure-guided screen of compounds from the FDA’s Generally Recognized as Safe (GRAS) list, selected for their commercial availability, low cost, and established safety profile, ensuring scalability and facilitating clinical translation. Candidate DBAs were first evaluated using *in silico* molecular docking to identify agents with strong predicted non-covalent binding affinity for TAC. Compounds exhibiting high-affinity interactions were then validated through *in vitro* release studies. Consistent with the modeling predictions, DBAs demonstrating stronger TAC binding, such as isoliquiritigenin and maltotriose, substantially reduced the initial burst release observed in DBA-free formulations. This effect was attributed to TAC-DBA interactions that slowed TAC diffusion and moderated solvent efflux, thereby stabilizing depot formation.

Co-encapsulation of rapamycin (RAPA) with TAC at optimized concentrations also attenuated the initial TAC burst release, suggesting that RAPA itself functions as a DBA through non-covalent interactions between the two macrolides. This is significant because, in clinical solid-organ transplantation (SOT), mTOR inhibitors such as RAPA or everolimus are often combined with TAC to achieve synergistic immunosuppression, resulting in lower acute rejection rates and mitigation of chronic vasculopathy and fibrotic remodeling (16–18). These findings demonstrate that the PRECISE platform can be readily configured for either single-agent TAC or dual-agent TAC+RAPA depots, enabling regimen-specific tailoring while maintaining precise release control through structure-guided co-formulation.

PRECISE was well tolerated *in vivo*, with complete resorption of the subcutaneously formed depot observed within one month of administration. In an orthotopic hindlimb transplantation model, repeated monthly injections of TAC and RAPA co-loaded PRECISE into the graft achieved prolonged, rejection-free graft survival for over 300 days, while maintaining systemic drug levels within the therapeutic window (5–10 ng/mL) and effectively suppressing the initial burst release. Immunological analysis revealed increased circulating regulatory T cells and reduced donor-specific lymphocyte proliferation compared to untreated controls, consistent with durable systemic immunoregulation mediated by sustained, precisely controlled immunosuppression.

In a porcine heterotopic gracilis flap transplantation model, injection of PRECISE into the graft prolonged survival beyond 90 days despite a full swine leukocyte antigen (SLA) Class I/II haplotype mismatch. In contrast, untreated controls exhibited complete graft rejection by postoperative day (POD) 5. The levels of systemic TAC and RAPA stayed in the therapeutic ranges throughout the study. Collectively, these results establish PRECISE as a clinically translatable platform capable of achieving long-acting and safe immunosuppression. The underlying design principles may also extend beyond VCA to solid-organ transplantation, where the depot can be placed peri-graft or administered remotely, such as subcutaneously in the abdomen, to achieve long-term immunosuppression with precisely controlled systemic drug levels.

## Results

### Incorporation of Structure-Guided DBAs into PLGA Depots Enables Controlled TAC Release

PLGA-based *in situ* forming depots have been widely explored for long-acting drug delivery and are used in several FDA-approved formulations due to their injectability, biocompatibility, and ability to provide sustained release through polymer degradation (19, 20). However, conventional PLGA depots often exhibit an initial burst release phase before reaching steady-state kinetics, which can lead to supratherapeutic drug exposure (21, 22). To address this limitation, we hypothesized that incorporating DBAs, small molecules capable of forming non-covalent interactions with TAC, could modulate TAC mobility within the depot, reducing burst release and enabling rapid establishment of a stable release rate. To test this concept, we first performed *in silico* molecular docking studies to evaluate the binding affinity of FDA’s GRAS-listed compounds with TAC. DBA candidates were then incorporated along with PLGA, and their *in vitro* TAC release profiles were compared to formulations lacking DBAs.

**Figure 2.**
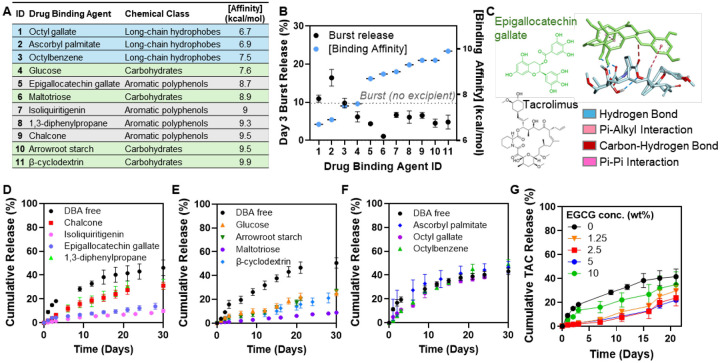
Structure-guided selection of DBAs attenuates tacrolimus burst from PRECISE depots. **(A)** Screen of GRAS-listed drug-binding agents (DBAs) across three chemical classes— aromatic polyphenols, carbohydrates, and long-chain hydrophobes—ranked by predicted TAC-binding affinities from AutoDock Vina (more-negative values indicate stronger predicted binding). **(B)** Plot of Day-3 cumulative TAC release (burst) versus predicted binding affinity for each candidate. Dashed line indicates burst from the no-excipient PRECISE control. **(C)** Representative docking of EGCG with TAC. TAC presents hydrogen-bond acceptors and π-rich, methylated surfaces that interact with the π-rich, hydrophobic EGCG via hydrogen bonds and π–π/π–alkyl interactions. **(D–F)** In vitro TAC release profiles in PBS containing 20% (v/v) methanol at 37 °C from PRECISE depots containing (D) aromatic polyphenols (e.g., EGCG, isoliquiritigenin, chalcone), (E) carbohydrates (e.g., maltotriose, β-cyclodextrin, arrowroot starch, glucose), and (F) long-chain hydrophobes (octylbenzene, ascorbyl palmitate, octyl gallate). DBA effects were statistically significant for polyphenols and carbohydrates. **(G)** In vitro TAC *cumulative* release profiles as a function of EGCG loading (0–10 wt%). Increasing EGCG reduced cumulative burst release up to 5 wt%, with a partial rebound at 10 wt%.

To guide DBA selection from the GRAS list, we analyzed the structural features of TAC to identify compounds capable of forming favorable non-covalent interactions. TAC is a highly lipophilic macrolide featuring a 23-membered macrocyclic lactone ring with multiple methyl substituents (e.g., Me 35, Me 39, Me 40, Me 42, Me 43) that make its surface strongly hydrophobic, lowering aqueous solubility. The molecule also contains three isolated C=C bonds (C19=C20, C27=C28, C37=C38) that contribute to a localized π-surface, and several oxygenated groups (carbonyl and ether oxygens) that can engage in hydrogen bonding and dipole–dipole interactions. These features suggested that candidate DBAs with complementary hydrophobic, π–π stacking, or hydrogen-bonding capabilities would be most effective in forming stable non-covalent TAC-DBA complexes to modulate drug release. Guided by this, we selected eleven GRAS-listed compounds spanning three broad chemical classes (Figure 2A): (i) aromatic polyphenols: epigallocatechin gallate (EGCG), chalcone, isoliquiritigenin, and 1,3-diphenylpropane, chosen for their π-rich, hydrophobic character; (ii) carbohydrates: β-cyclodextrin, maltotriose, arrowroot starch, and glucose, selected for their ring-based structures, which offer partial conformational compatibility with TAC’s macrocyclic core, and for their abundant hydroxyl groups capable of multivalent hydrogen bonding; and (iii) long-chain hydrophobes: ascorbyl palmitate, octyl gallate, and octylbenzene, selected for their flexible aliphatic tails, which can engage in hydrophobic interactions across TAC’s extensive non-polar surface. We then performed computational docking studies (AutoDock Vina) and analyzed TAC-DBA interaction geometries in PyMOL and BIOVIA Discovery Studio.

Docking analyses revealed distinct TAC-DBA interaction profiles across the eleven candidates (Figure 2A). Binding affinities ranged from –6.7 to –9.9 kcal/mol, with more negative values indicating stronger, more favorable interactions. Complementary *in vitro* release studies were then conducted to experimentally examine whether the interactions between TAC and DBAs could contribute to reducing burst release and extending sustained release relative to the DBA-free formulation. All formulations were prepared using 45 wt% PLGA dissolved in N-methyl-2-pyrrolidone (NMP), a biocompatible solvent, to which 5 wt% of the selected DBA was added relative to the total formulation. TAC was encapsulated at a concentration of 28 mg/mL, selected to deliver a 7 mg TAC dose in a 250 μL injection volume for in vivo studies, a dosing regimen that was effective in our previous works (23). We particularly paid attention to the cumulative drug release by Day 3 as a parameter representing burst release. DBAs with more favorable (i.e., more negative) binding energies generally reduced the initial burst release compared to the excipient-free formulation (Figure 2B). DBAs exhibiting stronger binding affinity suppressed TAC release more effectively than those with weaker interactions. However, the relationship between burst release and binding affinity was not strictly monotonic, suggesting that additional factors, such as NMP efflux, overall formulation hydrophilicity/hydrophobicity and pore size may also influence release kinetics.

Polyphenol-based excipients, including epigallocatechin gallate (EGCG), isoliquiritigenin, and chalcone, reduced burst release and overall release rate, as compared to the DBA-free control (Figures 2C and D), likely due to their aromatic, π-rich structures. EGCG emerged as a representative example (Figure 2C), exhibiting a dual benefit of strong molecular binding (−8.7 kcal/mol) and a greater than 50% reduction in burst release. Molecular docking showed that EGCG donates hydrogen bonds via its phenolic hydroxyls to TAC’s polar atoms (Figure S1), while π–π stacking with TAC’s alkene segments and π–alkyl interactions with methylated regions further stabilized the complex, suggesting dual hydrophilic and hydrophobic engagement.

All carbohydrate-based excipients tested, including maltotriose, β-cyclodextrin, arrowroot starch, and glucose, also suppressed burst release and modestly extended sustained release (Figures 2B and E). Maltotriose, a linear trisaccharide, showed the strongest effect, lowering Day 3 cumulative release to ~1%, compared to ~10% in DBA-free control. Docking analysis revealed that maltotriose engages in multiple conventional hydrogen bonds with TAC’s carbonyl and ether oxygen atoms (Figure S1), as well as carbon–hydrogen interactions with methyl-rich regions on the TAC surface. One unfavorable acceptor–acceptor contact was also observed but did not appear to disrupt overall affinity, which reached −8.9 kcal/mol.

Excipients with long aliphatic chains had a minimal effect (Figures 2B and F). Octylbenzene and ascorbyl palmitate, which exhibited the weakest binding affinities to TAC at −6.7 and −6.9 kcal/mol respectively, formed only limited non-polar contacts. The docking visualizations showed that their interactions were largely confined to van der Waals and nonspecific hydrophobic contacts with TAC’s aliphatic regions, and both compounds slightly increased burst release relative to the excipient-free control (Figure S1). This limited interaction profile may explain their poor performance in controlling initial drug diffusion, underscoring the importance of specific binding modes beyond general hydrophobicity.

Next, we evaluated whether the release-suppressing effect of DBAs was dependent on their concentration (Figure 2G). EGCG was selected as a representative DBA for this analysis. Cumulative TAC release decreased progressively with increasing EGCG concentrations up to 5 wt%, indicating enhanced drug retention within the depot. However, further increasing the EGCG content from 5 to 10 wt% led to a partial rebound in TAC release, although overall release remained lower than that of the DBA-free formulation.

In addition to directly interacting with TAC, DBAs also modulated solvent dynamics during depot formation. Both EGCG and β-cyclodextrin slowed NMP diffusion relative to the DBA-free formulation, thereby reducing the rapid solvent efflux that typically drives the initial burst release (Figure S2A). During the first 24 hours, NMP release from EGCG-containing PRECISE formulations was comparable to that of excipient-free controls. However, beyond this point, EGCG markedly slowed cumulative NMP efflux, with 56.3 ± 4.9% of solvent released by 96 hours compared to 96.3 ± 1.4% in controls.

Formulations incorporating these optimized DBAs were designated as PRECISE depots. The PRECISE platform thus represents an optimized, PLGA-based in situ forming depot that integrates structure-guided excipient selection to achieve precisely controlled, long-acting drug release.

### RAPA Functions as a DBA to Control TAC Release and Enable a Combination Immunosuppression Strategy

**Figure 3.**
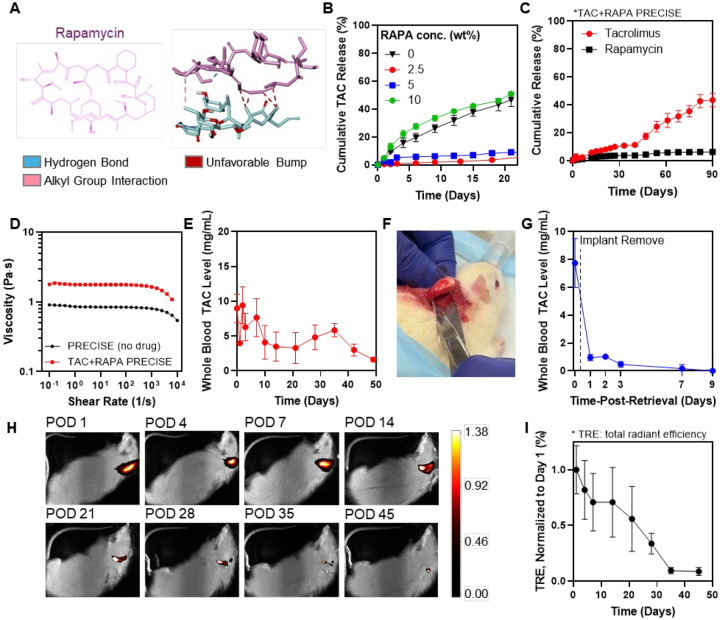
Rapamycin co-encapsulation suppresses tacrolimus burst and enables sustained, retrievable dual-drug delivery from PRECISE depots. **(A)** Representative docking of RAPA with TAC. RAPA, a rigid macrolide bearing methylated alkenes and hydrogen-bond donors, forms hydrogen bonds and π–π/π–alkyl interactions with TAC (predicted ΔG = −12.8 kcal/mol), suggesting stable multimodal complexation. **(B)** Cumulative TAC release from PRECISE with increasing RAPA loading (0, 2.5, 5, 10 wt%). TAC release is attenuated up to 5 wt% RAPA, with partial rebound at 10 wt%. **(C)** In vitro release profile of TAC and RAPA from PRECISE with 5 wt% RAPA. Both drugs exhibit low, sustained cumulative release over 3 months. **(D)** Viscosity of PRECISE with and without TAC and RAPA (rotational rheometry). Both formulations fall within the injectable range of commercial in situ–forming systems. **(E)** TAC pharmacokinetics in healthy rats after subcutaneous TAC+RAPA PRECISE injection, showing minimal burst and sustained systemic concentrations within the therapeutic range. **(F)** Photograph showing surgical retrieval of a single PRECISE depot. **(G)** TAC blood levels in rats decline rapidly following PRECISE retrieval, demonstrating controllable drug offloading. **(H)** Representative IVIS images of Cy7-labeled PLGA depots subcutaneously injected into rats. **(I)** Normalized total radiant efficiency from IVIS shows progressive signal loss with near-complete disappearance by 5–6 weeks, indicating full depot biodegradation.

Since RAPA, another widely used immunosuppressive agent, possesses a rigid macrolide ring (Figure 3A) decorated with multiple methyl groups and isolated double bonds, we hypothesized that it might also function as an effective DBA through non-covalent interactions with TAC. TAC and RAPA are frequently co-administered in transplant medicine (16–18), where their complementary mechanisms, calcineurin inhibition by TAC and mTOR pathway suppression by RAPA, yield synergistic immunosuppression that improves graft survival while reducing calcineurin-inhibitor–related toxicity (e.g., nephrotoxicity) relative to monotherapy (24). Accordingly, the co-encapsulation of TAC and RAPA within the PRECISE platform was designed to leverage their complementary mechanisms within a single depot, while enabling controlled, localized immunosuppression through RAPA’s additional role as a DBA.

*In silico* docking studies indicated that RAPA’s macrolide ring nests against TAC’s hydrophobic surface and π-rich alkene domains (Figure 3A). Simultaneously, RAPA’s hydroxyl and carbonyl groups form conventional hydrogen bonds with TAC’s carbonyl and ether oxygens, yielding a binding affinity of −12.8 kcal/mol. These multimodal contacts suggest that TAC-RAPA pairing could stabilize both molecules within the PRECISE depot.

Consistent with the *in silico* predictions, incorporation of RAPA into the formulation reduced TAC burst release *in vitro* in a concentration-dependent manner (Figure 3B). At 2.5 wt% RAPA, the Day-3 burst release decreased approximately fivefold, from ~10% in the RAPA-free control to ~2%, indicating enhanced stabilization of TAC within the depot. However, at higher RAPA loading (10 wt%), burst release rebounded, surpassing that of the RAPA-free control, a biphasic trend similar to that observed with EGCG. Based on these findings, 2.5 wt% RAPA was selected for subsequent dual-drug PRECISE studies, which translates to 24 mg RAPA/mL. In this optimized TAC+RAPA co-loaded formulation, both TAC and RAPA were released in a sustained, coordinated manner without any early burst phase (Figure 3C).

The TAC+RAPA co-loaded formulation also exhibited slower diffusion of NMP relative to the RAPA-free formulation (Figure S2A). By 96 hours, 71.4 ± 5.2% of total NMP was released from TAC/RAPA co-loaded depots, as compared to 95.5 ± 0.3 % from RAPA-free controls. This attenuated NMP efflux may also partly contribute to the reduced early burst phase, reinforcing the role of RAPA in modulating both drug–drug interactions and solvent dynamics during matrix formation.

Next, we evaluated the injectability of the PRECISE formulation using rotational rheometry across shear rates of 0.1–10^4^ s^−^¹. The viscosity of drug-free PRECISE was 0.97 ± 0.21 Pa·s, which increased to 1.76 ± 0.08 Pa·s upon co-loading with TAC and RAPA. Both values fall within the viscosity range reported for clinically used *in situ* forming depot systems such as Atrigel^®^ (~1–2 Pa·s) (Figure 3D) (25), which are compatible with syringe-based administration through 18–25 G needles. This underscores the translational potential of PRECISE.

To assess in vivo PK of TAC+RAPA co-loaded PRECISE, 0.25 mL of the formulation (7 mg TAC, 6 mg RAPA per animal) was injected subcutaneously into healthy rats, and whole-blood TAC concentrations were measured for 49 days. Following a small early rise (9.0 ± 5.0 ng/mL at Day 0), TAC showed a transient maximum of 9.4 ± 4.5 ng/mL on Day 2 and then stabilized in the ~3.0–6 ng/mL range through Day 49 (Figure 3E). Concentrations remained <10 ng/mL at all time points, indicating low-amplitude, sustained systemic exposure without supratherapeutic spikes.

To assess retrievability, we subcutaneously injected TAC+RAPA PRECISE into healthy rats and, after 7 days, surgically removed a single PRECISE depot under brief anesthesia and then monitored TAC levels post-excision. The depot was removed intact, as a single discrete mass, without fragmentation (Figure 3F), and circulating TAC declined sharply, falling from 7.75 ± 1.76 ng/mL at the time of excision (Day 0) to 0.95 ± 0.23 ng/mL at 24 h (~90% decrease). On day 9 following the retrieval, the values approached the assay’s lower quantification limit of 0.16 ng/mL (Figure 3G). These data demonstrate that PRECISE enables rapid reduction of systemic TAC exposure, a safety capability not available with other injectable implant platforms such as dispersed microspheres or infiltrating hydrogels, which are not practically retrievable. This reversibility supports clinician-directed cessation or dose reduction when toxicity, infection, or other clinical considerations arise.

To confirm depot degradation, PLGA was labeled with a near-infrared Cy7 fluorophore and tracked *in vivo* using IVIS imaging. The subcutaneously injected depot exhibited a gradual decline in fluorescence intensity over time, with near-complete signal loss by 5–6 weeks, indicating complete degradation of the polymeric matrix (Figures 3H,I). This degradation profile and the PK data support the feasibility of monthly to bimonthly dosing intervals for sustained drug delivery.

### PRECISE Enables Sustained Immunosuppression and Long-Term Graft Survival in a Rat Hindlimb Transplant Model

**Figure 4.**
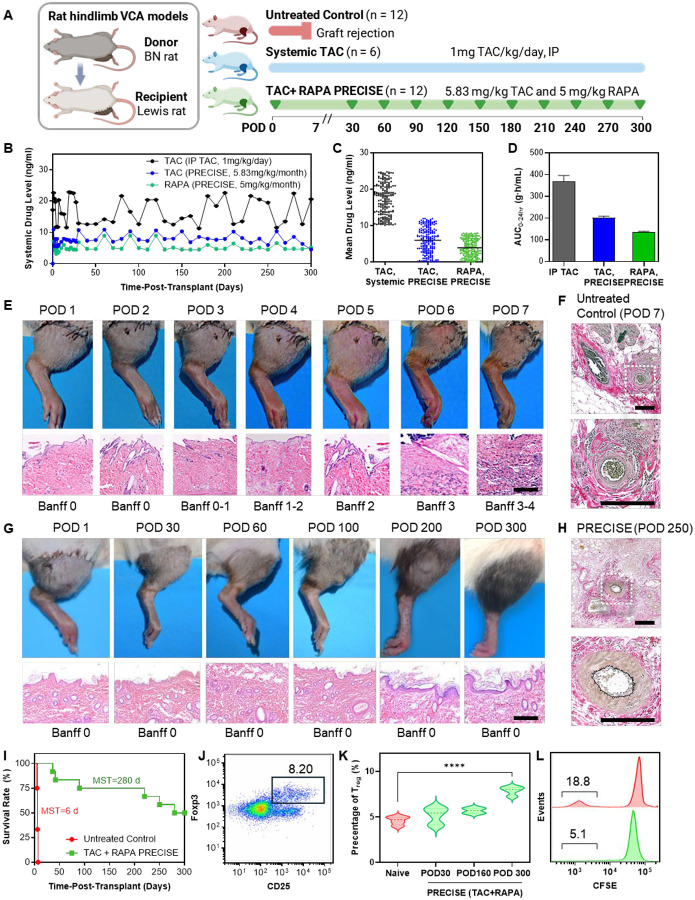
In vivo evaluation of dual-drug PRECISE in a rat limb transplant model. **(A)** Schematic of the study design. Orthotopic hindlimb transplants were performed from Brown Norway (BN) donors to Lewis recipients. Animals received either no treatment, daily intraperitoneal (IP) TAC (1 mg/kg), or monthly subcutaneous PRECISE injections (0.25 mL, 7 mg/mL TAC + 6 mg/mL RAPA). **(B)** Wholeblood drug levels over time in systemic TAC–treated (black, TAC) and PRECISE–treated (blue, TAC; green, RAPA) animals. PRECISE maintained therapeutic levels (5–10 ng/mL) with minimal fluctuation. **(C)** Beeswarm plots showing cumulative whole-blood exposure to TAC and RAPA over the 300-day study period. Systemic TAC treatment resulted in higher and more variable cumulative TAC levels compared to PRECISE, while PRECISE maintained lower, more consistent exposure to both drugs. **(D)** Early systemic exposure measured by AUC₀–24 h following a single dose. TAC AUC was lower in PRECISE vs. systemic TAC; RAPA AUC shown for dual-drug PRECISE. **(E)** Time-series images showing progressive rejection in untreated recipients, with blackened digits and skin necrosis. H&Estained tissue sections from untreated controls showing severe acute rejection and necrotic tissue (scale bar: 200 μm). **(F)** Verhoeff–Van Gieson elastin staining in untreated animals on POD 7 showing perivascular infiltration and preserved elastic laminae without intimal thickening (scale bar: 200 μm). **(G)** Time-series images from PRECISE–treated recipients showing graft survival, healing, and full fur regrowth by POD 300. H&E sections from PRECISE–treated animals showing preserved dermal and epidermal architecture at late timepoints (scale bar: 200 μm). **(H)** Elastin staining in PRECISE–treated grafts on POD 250 showing patent vessels with intact elastic laminae and no signs of vasculopathy (scale bar: 200 μm). **(I)** Kaplan–Meier survival curve of untreated and PRECISE-treated recipients post-transplant. All untreated grafts were rejected by POD 7 (median survival: 6 days). PRECISEtreated animals showed median survival >250 days, with 6 of 12 maintaining viable grafts through POD 300. **(J)** Representative flow cytometry showing CD4^+^CD25^+^Foxp3^+^ regulatory T cells (Tregs) from a PRECISE-treated animal on POD 300. **(K)** Violin plots showing longitudinal increase in circulating Tregs in PRECISE-treated animals at POD 30, POD 160, and POD 300 compared to naïve controls. **(L)** CFSE-based MLR showing reduced donor-specific lymphocyte proliferation in PRECISEtreated animals vs. naïve controls, consistent with donor-specific hyporesponsiveness.

To evaluate the immunosuppressive efficacy of the TAC+RAPA co-loaded PRECISE formulation in preventing graft rejection, we used a rat orthotopic hindlimb allotransplantation model (Figure 4A). Transplants were performed from Brown Norway (BN) donors to Lewis recipients, which carry distinct RT1 haplotypes, RT1^n^ and RT1^l^, respectively, that differ across multiple genes within the RT1 complex (26, 27). These genetic disparities result in strong incompatibility at the level of antigen presentation, making this model a stringent test of immunosuppressive efficacy.

Three treatment groups were evaluated: (i) untreated controls, (ii) a systemic TAC comparator group receiving once-daily intraperitoneal (IP) TAC at 1 mg/kg/day, and (iii) the TAC+RAPA co-loaded PRECISE group. In the PRECISE group, animals received a 0.25 mL subcutaneous depot injection at the time of surgery (POD 0), divided between the dorsal and ventral aspects of the transplanted limb. Each depot contained 28 mg/mL TAC and 24 mg/mL RAPA, delivering 7 mg TAC and 6 mg RAPA per animal (5.83 mg/kg TAC; 5.00 mg/kg RAPA). Monthly maintenance injections of the same volume and composition were administered through POD 300, for a total of 11 doses per animal.

Because the first dose was applied directly onto the wound bed during surgery, it was essential that the formulation remain localized rather than spreading across surrounding tissue, which would increase exposed surface area and alter release kinetics. This required a shear-thinning system, one that flows readily during injection but rapidly regains viscosity afterward to form a stable depot. To achieve this, 5 wt% palm oil (PO) was added to the standard NMP/PLGA formulation (40 wt% PLGA, 55 wt% NMP, 5 wt% PO), and TAC was pre-encapsulated within a lyophilized triglycerol monostearate (TG-18) hydrogel (80 mg/mL) (23) and then incorporated directly into the PLGA phase. Palm oil, a viscous and hydrophobic excipient, was expected to increase overall formulation viscosity and reduce solvent mobility, thereby limiting flow at the injection site. TG-18, a low-molecular-weight amphiphilic gelator, self-assembles through hydrogen bonding and hydrophobic interactions into a weak supramolecular network that behaves as a viscoelastic solid at rest. Under shear, this non-covalent network partially disrupts, leading to a reversible decrease in viscosity that facilitates injection; once shear is removed, the network rapidly reforms, allowing the formulation to re-solidify into a cohesive depot (28). These modifications increased formulation viscosity while imparting desirable shear-thinning behavior (Figure S3A). Because the TG-18 matrix transiently entraps TAC through weak non-covalent interactions, released TAC can still associate with RAPA in the surrounding PLGA phase, preserving the coordinated, controlled release profile characteristic of PRECISE. Consistently, the modified formulation containing palm oil and TG-18 hydrogel exhibited release kinetics comparable to those of the original PRECISE formulation lacking these additives (Figures S3B, C). Accordingly, this optimized, shear-thinning formulation was used for all subsequent *in vivo* studies.

In the control group receiving daily systemic TAC injections, animals exhibited high and variable blood concentrations throughout the study, with mean levels fluctuating around 19.3 ± 3.8 ng/mL and repeated supratherapeutic peaks exceeding 22 ng/mL at several timepoints (e.g., Days 1, 19, 120, and 200) (Figures 4B, C). These fluctuations highlight the challenge of maintaining stable systemic exposure with bolus dosing. In contrast, in transplant recipients treated with the PRECISE, TAC levels were 10.6 ± 0.64 ng/mL on Day 1, without a discernible post-injection spike, and remained within the 5–10 ng/mL therapeutic range through Day 300 despite repeated monthly injections. RAPA exhibited a similar trend, reaching 7.1 ± 0.61 ng/mL on Day 1 and stabilizing near 5 ng/mL thereafter.

To assess early systemic exposure, we calculated AUC_0–24 h_ for TAC and RAPA using the trapezoidal rule based on plasma concentrations collected from 0 to 24 hours (3-hour intervals) following administration of either PRECISE or systemic TAC (Figures 4D, S4). Early systemic exposure was 368.60 ± 27.11 ng·h/mL for systemic TAC, 201.11 ± 7.47 ng·h/mL for TAC released from PRECISE, and 134.24 ± 5.22 ng·h/mL for RAPA released from PRECISE. Relative to systemic TAC, PRECISE reduced TAC AUC_0–24 h_ by more than 40%. These data indicate that the local depot moderates initial systemic exposure while maintaining therapeutic blood levels over an extended period, consistent with the goal of achieving precise and sustained drug delivery with minimized peak fluctuations.

We also evaluated allograft survival to determine the *in vivo* immunosuppressive efficacy of the PRECISE formulation. In untreated controls, all grafts were rejected within 7 days post-transplantation, with a median survival of 6 days (Figures 4E, I). Histopathological analysis of grafts collected at POD 7 revealed severe acute rejection, with Banff grades 3–4 (29), extensive tissue necrosis, dense mononuclear infiltration, and vascular injury on hematoxylin and eosin (H&E) staining. Macroscopic examination showed blackened digits and circumferential skin sloughing, consistent with advanced rejection. Verhoeff–Van Gieson (VVG) elastin staining further demonstrated prominent perivascular inflammatory cuffs surrounding vessels with preserved elastic laminae, indicative of perivasculitis without significant intimal thickening (Figure 4F).

In contrast, PRECISE-treated animals exhibited markedly extended graft survival (Figures 4G, I). Six of twelve recipients maintained viable grafts throughout the full 300-day study period with no evidence of rejection, and the median survival in the PRECISE group exceeded 250 days, representing more than a forty-fold increase compared to controls. Treated animals showed progressive healing, with sustained limb function, normal skin coloration, and complete fur regrowth over time (Figure 4G), consistent with long-term graft viability. Consistent with these outcomes, Verhoeff–Van Gieson elastin staining at POD 250 in PRECISE-treated recipients demonstrated patent vascular lumina with continuous elastic laminae, no perivasculitis, and no intimal hyperplasia or graft vasculopathy (Figure 4H).

To assess immune regulation associated with long-term graft survival, we performed longitudinal flow cytometric analyses of peripheral blood mononuclear cells (PBMCs) in PRECISE-treated recipients versus naïve Lewis rats. Regulatory T cells (Tregs) were defined as CD4^+^CD25^+^Foxp3^+^ populations, using a sequential gating strategy (Figure S5). Compared to naïve Lewis rats, PRECISE-treated recipients exhibited elevated frequencies of circulating Tregs at multiple time points post-transplantation. The proportion of CD4^+^ T cells expressing CD25 and Foxp3 increased from a baseline of 4.2 ± 0.4% in naïve animals to 5.9 ± 0.6% at POD 30, 6.3 ± 0.5% at POD 160, and 8.5 ± 0.4% at POD 300 (Figures 4J, K). These data indicate an expansion of the Treg compartment in the peripheral blood over time, consistent with a state of active immune regulation in long-term allograft recipients.

To evaluate donor-specific immune reactivity, we performed mixed lymphocyte reaction (MLR) assays using PBMCs isolated from PRECISE-treated recipients at POD 300 and naïve Lewis controls. Responder cells were labeled with carboxyfluorescein succinimidyl ester (CFSE) and co-cultured with irradiated splenocytes from donor BN rats as stimulators. Flow cytometric analysis of CFSE dilution showed markedly reduced lymphocyte proliferation in long-term recipients compared with naïve animals, with 5.1% versus 18.8% of CFSE^+^ cells undergoing division, respectively (Figure 4L). This attenuated proliferative response indicates donor-specific hyporesponsiveness in long-term PRECISE-treated recipients, suggestive of immunologic tolerance or sustained suppression.

Taken together, these findings demonstrate that the PRECISE formulation achieves sustained release of both TAC and RAPA with systemic concentrations maintained within the therapeutic window, resulting in prolonged graft survival and long-term immune regulation characterized by Treg expansion and reduced donor-specific alloreactivity.

### In Vivo Large Animal Evaluation of PRECISE in a Porcine Flap Transplant Model

**Figure 5.**
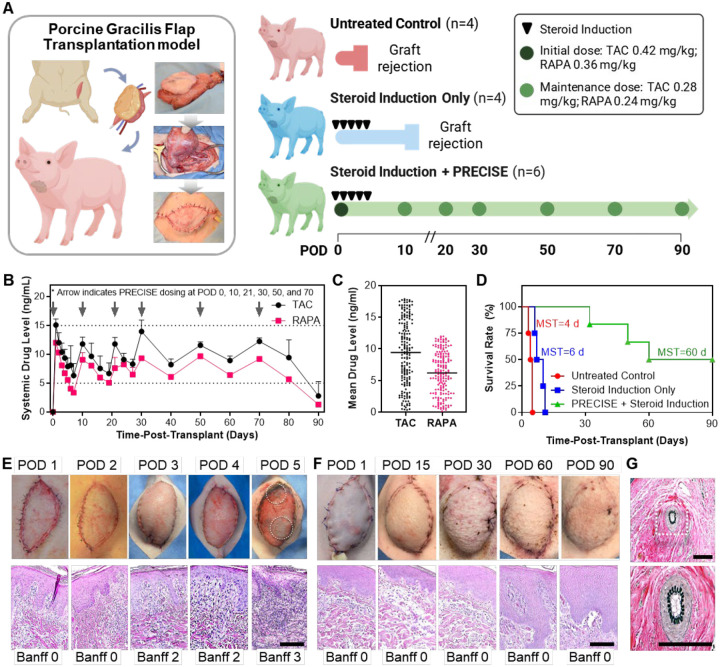
PRECISE enables sustained systemic immunosuppression and prolonged flap survival in a stringent porcine VCA model. **(A)** Schematic of the study design. Myocutaneous gracilis flaps were transplanted between fully mismatched miniature swine donor–recipient pairs. Animals received either no treatment, daily steroid induction only, or steroid induction plus monthly subcutaneous PRECISE injections. **(B)** Whole-blood drug levels over time in PRECISE–treated animals (black, TAC; magenta, RAPA). PRECISE maintained both agents within or near the therapeutic window (TAC 5–15 ng/mL, RAPA 5–10 ng/mL) with gradual decline. **(C)** Bee swarm plots showing cumulative systemic exposure to TAC and RAPA over the 90-day study period in PRECISE-treated animals. **(D)** Kaplan–Meier survival curve of flap grafts. Untreated and steroid-only grafts were rejected by POD 5 and 11, respectively, while PRECISE-treated grafts remained viable in 3 of 6 animals through POD 90. **(E)** Gross images and H&E-stained sections from untreated and steroid-only animals showing progressive epidermal necrosis and severe acute rejection by POD 5 (scale bar: 200 μm). **(F)** Gross and histological appearance of PRECISE-treated grafts showing intact architecture and complete fur regrowth through POD 90 with minimal inflammation (scale bar: 200 μm). **(G)** Verhoeff–Van Gieson elastin staining in PRECISE-treated grafts at POD 90 revealing patent vessels with intact elastic laminae and no evidence of vasculopathy (scale bar: 200 μm).

To assess PRECISE in a clinically relevant large-animal model, we performed allogeneic myocutaneous flap transplantation in miniature swine (mean body weight approximately 25 kg; gracilis free-flap weight approximately 200 g) under conditions of maximal immunological challenge (Figure 5A). Donor-recipient pairs were selected based on a compatibility matrix designed to maximize immunologic disparity in their major histocompatibility complex (MHC) antigens while maintaining ABO compatibility (Figure S6). Porcine MHC, or the swine leukocyte antigen (SLA), genotyping was performed as previously described (PMID 19392823 and 20121817) with modifications made to the typing primer panels to broaden allele coverage and enhance typing resolution in outbred farm pigs. ABO-incompatible pairs were excluded to prevent hyperacute rejection. Pairs with closely matched SLA were excluded to avoid immunologically permissive transplants. Final selections of donor-recipient pairs prioritized full class I (SLA-1, SLA-2, SLA-3) and class II (DRB1, DQB1) haplotype and antigen mismatches to promote strong alloreactivity. Two reciprocal donor-recipient combinations were selected to control for immunological directionality and to evaluate PRECISE under the most stringent allogeneic conditions.

We evaluated three cohorts under escalating immunosuppressive conditions: (i) untreated control, (ii) systemic steroid induction only, and (iii) TAC+RAPA co-loaded PRECISE plus systemic steroid induction. The steroid induction regimen comprised intraoperative Solu-Medrol (500 mg IV), followed by a once-daily stepwise decrease of 100 mg on postoperative days 1–4, after which steroids were discontinued. PRECISE dosing in the porcine model was determined a priori using a target-tissue scaling framework derived from the rat hindlimb study. The rat formulation achieved therapeutic exposure at a 5.8 mg/kg TAC and 5.0 mg/kg RAPA dose. To translate this to the larger porcine gracilis flap, we normalized by the depot surface-area-to-tissue-mass ratio and applied a conservative downward adjustment to account for slower drug clearance in swine. TAC and RAPA have half-lives of 6–8 hours and 12–20 hours in pigs, respectively, compared with 1–2 hours and ~6 hours in rats. This scaling approach guided the final induction and maintenance doses. In the PRECISE cohort, PRECISE was administered as follows: at transplantation (induction), six 0.25 mL injections (total 1.5 mL; 7.0 mg/mL TAC and 6.0 mg/mL RAPA) delivering a total of 10.5 mg TAC and 9 mg RAPA per animal (0.42 mg/kg TAC; 0.36 mg/kg RAPA). Maintenance doses were given on POD 10, 21, 30, 50, and 70, each consisting of four 0.25 mL injections (total 1.0 mL) delivering 7 mg TAC and 6 mg RAPA per animal (0.28 mg/kg TAC; 0.24 mg/kg RAPA). Injections were distributed across the flap, with three deposits placed subcutaneously and three intramuscularly at the flap corners. Over the 90-day study, the cumulative doses were 45.5 mg TAC and 39 mg RAPA per animal.

Systemic drug levels were quantified in the PRECISE plus steroid cohort. TAC levels reached 15.08 ± 1.02 ng/mL on POD 1 and were subsequently maintained within 6.33–15.08 ng/mL for the majority of the 90-day observation period (Figure 5B). RAPA exhibited an initial concentration of 12.01 ± 0.17 ng/mL on POD 1, followed by sustained levels of 5.57–10.11 ng/mL through most of the study and a gradual decline to 1.29 ± 0.05 ng/mL by POD 90. These data demonstrate that the PRECISE regimen provided prolonged systemic exposure to both immunosuppressants, with concentrations maintained within their respective therapeutic ranges for extended periods (Figure 5C).

In the absence of immunosuppression, all grafts failed rapidly, with loss of perfusion at POD 5 (Figure 5D). In the steroid-only cohort, all grafts were rejected by POD 11, and survival lasted from POD 6 to POD 11. In the PRECISE plus steroid cohort, all grafts remained viable through POD 32, five of six remained viable at POD 50, four of six at POD 60, and three of six at POD 90. The histopathology corroborated the survival findings. Control grafts sampled at POD 5 demonstrated severe acute rejection (Banff 3–4) with dense mononuclear infiltration, vascular injury, and dermal–epidermal necrosis (Figures 5E). In the PRECISE plus steroid cohort, specimens collected at POD 90 retained dermal and epidermal architecture, showed minimal inflammatory infiltrates, and exhibited preserved vascular integrity, consistent with Banff 0–1 (Figures 5F). Gross images documented early epidermal necrosis and circumferential skin sloughing in controls and showed progressive healing, restoration of normal skin coloration, and complete hair regrowth in the PRECISE–treated cohort. To further assess vascular integrity, Verhoeff–Van Gieson elastin staining in treated recipients at POD 90 revealed intact elastic laminae, open lumens, and an absence of perivascular inflammation or vasculopathy, indicating preserved vascular architecture without evidence of chronic rejection (Figure 5G).

Flow cytometry of PBMCs in the porcine PRECISE + steroid cohort showed a progressive rise in circulating regulatory T cells (CD4^+^CD25^+^Foxp3^+^), increasing from ~5% at POD 0 to ~11% by POD 80 (Figure S7). To enable terminal tissue collection while minimizing repeated invasive sampling, we used a cross-sectional, serial-sacrifice design with one animal per time point, consistent with the principles of Replacement, Reduction, and Refinement (3Rs) and the ARRIVE 2.0 guidelines. Accordingly, these data are descriptive and not powered for statistical (longitudinal) inference; confirmation in larger longitudinal cohorts is needed. These descriptive data are directionally consistent with the rat results and with the extended graft survival observed in this study.

## Discussion

VCA remains constrained by toxicities and the variations in systemic levels of orally administered calcineurin-inhibitor–based regimens such as TAC. TAC has a narrow therapeutic window (~5–10 ng/mL), exhibits high inter- and intrapatient pharmacokinetic variability from absorption and CYP3A metabolism, and therefore requires intensive therapeutic drug monitoring; excursions above and below target are linked to nephrotoxicity or infection risk and rejection, respectively. (6, 30, 31) In this study, we present PRECISE, a graft-embedded, long-acting formulation that enables precise and sustained immunosuppression through structure-guided control of TAC release. By incorporating DBAs that form non-covalent interactions with TAC, PRECISE maintains drug concentrations stably within the therapeutic window while eliminating the burst release that limits conventional depots. RAPA also functions as a DBA, and when co-formulated with TAC at optimized ratios, it not only regulates TAC release kinetics but also provides complementary mTOR-mediated immunomodulation, resulting in durable graft protection. The *in situ* forming injectable depot maintained stable systemic TAC and RAPA levels and prolonged graft survival in VCA models, exceeding 300 days in rats and 90 days in pigs, without clinical or histologic evidence of rejection. To our knowledge, the current study represents the first demonstration of a combination-immunosuppressant depot capable of sustaining long-term VCA graft survival in the absence of continuous systemic therapy with systemic drug levels precisely maintained within the therapeutic window.

Various drug-delivery systems have been developed as carriers for long-acting immunosuppressive therapy in VCA, ranging from solvent-based intragraft drug solutions (12) and hydrogels (11, 13, 23) to microspheres/nanoparticles (18, 19). PRECISE offers several key advantages over these earlier approaches. It effectively prevented burst release and established stable, steady-state blood levels shortly after administration. Many prior systems on the other hand, exhibit substantial burst release and limited control over long-term kinetics (13, 14). For example, a triglycerol monostearate (TG-18) hydrogel previously developed by our group achieved long-term TAC release and extended VCA survival beyond 100 days in rats following a single injection. (23) However, this system produced an initial mean blood TAC concentration of ~127 ng/mL, an order of magnitude higher than the ~10 ng/mL observed with PRECISE, before gradually stabilizing. In the porcine model, TG-18 similarly extended graft survival to 90 days but exhibited an initial peak of ~40 ng/mL, whereas PRECISE maintained an initial level near 15 ng/mL and reached near–steady-state plasma concentrations from day 1. In contrast, the TG-18 hydrogel required a prolonged equilibration period in rats and failed to achieve steady-state levels in pigs.

Another group reported a single injection of PLGA microspheres co-encapsulating TAC with mycophenolate mofetil and prednisone, which prolonged median graft survival to >150 days in a rat model of hindlimb transplantation (32). However, this formulation exhibited an excessive initial burst (>200 ng/mL) and failed to achieve a steady-state plasma concentration, with levels declining to ~10 ng/mL only after two months. Together, these comparisons highlight the distinct pharmacokinetic precision and safety profile of PRECISE, which rapidly achieves and maintains stable therapeutic drug levels while eliminating burst-associated toxicity.

Moreover, the ability of PRECISE to co-encapsulate TAC and RAPA in a single injectable depot formulation is an important step toward clinical translation of combination immunosuppression. Prior long-acting strategies in VCA have been mostly limited to single-agent delivery. PLGA microspheres encapsulating TAC/MMF/prednisone prolonged survival, yet key mechanistic questions were not addressed. In particular, drug–drug and drug–polymer interactions that dictate co-diffusion and burst were not characterized, and relative release rates across agents were not delineated to establish synchronous versus sequential exposure. In addition, these formulations exhibited a pronounced early systemic burst with high peaks and a slow approach to steady-state, and because the particles are dispersed, they are not practically retrievable once injected, which constrains rapid dose reduction in the event of toxicity. By contrast, PRECISE enables coordinated, sustained release of a calcineurin inhibitor (TAC) and an mTOR inhibitor (RAPA), dampens early burst, permits surgical retrieval of the depot, and aligns with clinical practice in which mTOR inhibitors are combined with calcineurin inhibitors to synergistically prevent rejection and limit chronic vasculopathy (33). Importantly, both drugs showed no burst release and rapidly achieved steady-state plasma concentrations within their therapeutic windows.

Furthermore, PRECISE is retrievable. Upon injection, the formulation undergoes phase inversion from liquid to solid, resulting in a localized implant that can be surgically excised if rapid drug cessation is required. By contrast, solvent-based intragraft solutions (e.g., DMSO-based) and dispersed microspheres/nanoparticles are not practically retrievable once injected, and many tissue-infiltrating injectable hydrogels such as TG-18 likewise cannot be removed without collateral tissue disruption. Given TAC’s narrow therapeutic window and risk of toxicity with supratherapeutic exposure, this capability for rapid discontinuation is a clinically important advantage of PRECISE. Consistent with this, depot excision in rats reduced circulating TAC from 7.75 ± 1.76 ng/mL at the time of removal to 0.95 ± 0.23 ng/mL at 24 h (~90% decrease), with values approaching the assay lower limit of quantification by Day 9, demonstrating practical, clinician-directed dose interruption when needed.

PRECISE achieved rapid steady-state drug levels and prevented burst release through structure-guided co-formulation of TAC with small-molecule DBAs. The DBAs formed non-covalent interactions with TAC that likely reduced its diffusivity within the polymer matrix, while their presence also modulated solvent efflux during phase inversion, possibly through physicochemical interactions with the solvent that stabilized depot formation. The effect of DBAs was concentration-dependent: for both EGCG and RAPA, we observed reduced burst release and slower overall release kinetics at 2.5–5 wt%, whereas the effect was lost at 10 wt%. We hypothesize that this non-monotonic behavior arises from excipient saturation within the polymer matrix or disruption of the depot microstructure at higher DBA loadings, which could diminish drug–DBA interactions or alter solvent efflux dynamics during depot formation.

Despite these encouraging results, our approach has certain limitations, necessitating additional studies to address remaining questions. First, the dosing intervals used here were intentionally conservative to prioritize graft stability rather than to define the maximal duration achievable with a single depot. In our models, TAC and RAPA depots were administered approximately once per month in rats and every 2–4 weeks in pigs to maximize graft success. Even these dosing intervals, every two weeks or once a month, would be a major improvement over the current daily oral regimen. Less frequent dosing may be feasible; future studies in healthy large animals should therefore quantify the duration of exposure following a single injection and define redosing triggers based on PK monitoring and early clinical or histologic indicators of rejection. If needed, formulation parameters, such as higher-molecular-weight PLGA, modified lactic:glycolic ratios, or increased depot volume, can be optimized to further extend dosing intervals while maintaining precise systemic control.

Second, additional studies on single-drug PRECISE depots co-formulated with DBAs would be valuable. We observed that RAPA itself functioned as a DBA, suppressing TAC burst release and promoting controlled, sustained delivery. Owing to this intrinsic effect, subsequent studies primarily focused on the combination formulation. Consequently, we did not perform detailed single-agent evaluations of TAC-only depots co-formulated with small-molecule DBAs such as EGCG or maltotriose, despite their excellent *in vitro* performance. Because many patients are maintained on TAC monotherapy in clinical practice, future work should investigate single-drug PRECISE depots with DBAs to better define *in vivo* dose-response relationships, release durability, and safety.

Third, while our miniature swine model offers clinically relevant skin and soft-tissue architecture under stringent histocompatibility mismatch, it does not fully recapitulate human pharmacokinetics, immunosurveillance, or clinical management. Studies in non-human primate (NHP) VCA models (for example, rhesus or cynomolgus macaques) could better align with human drug metabolism and therapeutic drug monitoring, and enable evaluation of steroid-sparing regimens, retrieval protocols, and long-horizon safety. These studies will help refine dose scaling, depot placement, and redosing triggers ahead of early-phase trials.

In summary, PRECISE provides a simple yet powerful strategy for achieving precise, sustained, and safe immunosuppression in VCA. By coupling TAC with structure-guided DBAs, PRECISE eliminates burst release, maintains therapeutic drug levels, even for combination immunosuppression, and enables long-term graft survival without continuous systemic dosing. The depot is also retrievable, offering an important safety feature for clinical translation. To our knowledge, this is the first demonstration of a combination-immunosuppressant depot capable of maintaining therapeutic precision and durable graft protection without daily systemic therapy.

## Materials and Methods

### Preparation of PRECISE

The pre-polymer solution was composed of 40 w/v % PLGA (L:G 85:15, 10–15 kDa, Poly Scientific) and TAC at a concentration of 28 mg/mL. Depending on the presence of excipients, the formulation contained either 55 v/v % NMP (Sigma-Aldrich) with 5 w/v % excipients or 60 v/v % NMP without excipients. All components were added to an 8 mL glass vial and heated until completely dissolved. The formulation was then drawn into a syringe and injected through 23G needle either into a sink consisting of 20% methanol in PBS for in vitro studies or subcutaneously into the transplantation site in animals for in vivo studies. Following administration, formulation underwent solvent exchange, polymerizing into a solid depot.

### In vitro drug release

100 uL of drug-loaded pre-polymer mixture (40w/w% PLGA, 55% or 60%w/w% NMP, and 28mg/ml TAC) was injected into the bottom of a 50mL Falcon tube using a syringe fitted with a 23G needle. Then the sink medium was added. For TAC-loaded PRECISE, we used 20% methanol in water as the sink medium due to low water solubility of the drug. The Falcon tube containing PRECISE depot was then incubated at 37 °C in a shaker incubator. The release medium was collected at pre-determined time points to be analyzed on HPLC.

The collected release sample was subjected to 24h freeze-drying to remove solvents and then reconstituted in 1ml ACN (Sigma-Aldrich). The drug concentration was determined using HPLC (Agilent 1260 Infinity II, USA) with a ZORBAX 300SB-C18 column (Agilent, 3.0 × 150 mm, 3.5 μm) at 40 °C. Chromatographic separation was achieved by gradient elution method, as described in Table S1. Calibration standards were prepared in the range of 0.78 – 400 mg/ml and cumulative drug release was calculated accordingly.

### Injectability characteristics

A rheometer equipped with a parallel plate with a diameter of 20 mm and a gap of 1 mm was used to determine the dynamic viscosity of the pre-polymer at room temperature. The solutions were pipetted onto the rheometer, and any excess solution was removed before measurement. The viscosity of the formulation was measured as the shear rate was swept from 1 to 1000 s^−1^. The force required to inject 1 mL of ISFI pre-polymer solution in 30 seconds was calculated using the Hagen-Pouiselle equation:

$$F=\frac{32\mu LQD^{2}}{d^{4}}$$

Where μ is the dynamic viscosity of ISFI formulation determined from the rheometer, L is length of needle (15 mm), Q is volumetric flow rate (2 mL/min), D is inner diameter of syringe barrel (8.66 mm), and d is inner diameter of needle (0.337 mm).

### In vitro degradation assessment

To assess in-vitro degradation of PRECISE, a weight loss assay was conducted. PRECISE pre-polymer mixture was injected into a 50 mL Falcon tube and weighed to determine the initial weight, followed by PBS addition as the sink medium. The samples were incubated in a shaker incubator at 37 °C. At designated time intervals (Day 1,2,4,7,14,28,30,42,60,80), the sink medium was removed, and samples were freeze-dried for 24 hours to remove any residual sink medium. The final weight of the dried samples were weighted, and the weight loss (degradation) was calculated as a percentage using the following equation:

$$\text{degradation}\;\%=\frac{W_{i}-W_{f}}{W_{i}}\times 100\%$$

Where *W*_*i*_ is the initial weight; *W*_*f*_ is the final weight after lyophilization.

### Molecular docking study

To evaluate the binding affinities and interactions between TAC and excipients containing long carbon chains, benzene rings, and heterocyclic rings, molecular docking simulations were performed using PyMOL 2.2.5, AutoDock Vina, and Biovia Discovery Studio 2021 Client. Molecular structures were sourced from the PubChem Database and the Protein Data Bank (RCSB PDB) and converted to .pdb format using PyMOL. These structures were then imported into AutoDock Vina for docking simulations, employing a grid box approach with dimensions of 15 × 15 × 15 Å along the X-, Y-, and Z-axes. Binding affinities, expressed in kcal/mol, were calculated by AutoDock Vina, which also generated complex files for each excipient-TAC interaction. Subsequent analyses of these complexes were conducted using Biovia Discovery Studio 2021 Client to identify and characterize specific interactions between TAC and the excipients.

### Animals

Male Lewis rats (200–250 g; 8–10 weeks old) were used as recipients for all VCA and pharmacokinetic studies. Brown Norway (BN) and Fischer 344 (F344) rats of similar age and weight served as donors for acute- and chronic-rejection models, respectively. Animals were maintained under controlled temperature, humidity, and a 12-hour light/dark cycle with ad libitum access to food and water. All procedures were approved by the IACUC and the Department of Defense ACURO and conformed to the NIH Guide for the Care and Use of Laboratory Animals.

Large-animal studies were performed in domestic crossbred swine (Sus scrofa domesticus; Landrace × Yorkshire × Duroc, “L × Y × D”; 6–8 weeks old; 15–20 kg) to capture natural outbred heterozygosity within the SLA complex. All porcine procedures were approved by the institutional IACUC and ACURO and performed under veterinary supervision in compliance with the Guide for the Care and Use of Laboratory Animals (8th edition).

### Pharmacokinetic sampling and drug quantification

For rats, blood was collected from the tail vein at pre-determined time points into EDTA-coated tubes and processed as whole blood. For swines, auricular-vein whole blood was sampled every 3–4 days during the first two postoperative weeks and every 6–10 days thereafter until day 90. Samples were kept on ice and aliquoted the same day; where applicable, additional aliquots were stored at −80 °C. Quantification of TAC and RAPA in whole blood (and, where specified, graft tissue) was performed using LC–MS/MS developed with the Wake Forest Proteomics and Metabolomics Shared Resource (adapted from Feturi, University of Pittsburgh PhD Thesis, 2020). For sample preparation, 20 μL of whole blood was mixed with 80 μL of extraction buffer containing 30 mM ZnSO_4_ and 10 ng/mL ascomycin (internal standard) in 80% methanol, vortexed, and centrifuged at 14,000 × g for 10 min at 4 °C; supernatants were analyzed directly. Calibration employed the Chromsystems 6PLUS1 Multilevel Whole Blood Calibrator Set for immunosuppressants.

Chromatography was performed on a Shimadzu Nexera UHPLC coupled to an AB Sciex Triple Quad 7500. Analytes were separated on a Phenomenex Kinetex C18 column (30 × 2.1 mm, 2.6 μm) with C18 guard at 60 °C; mobile phase A was 2 mM ammonium acetate with 0.1% formic acid in water, and mobile phase B was 2 mM ammonium acetate with 0.1% formic acid in methanol; flow 0.65 mL/min; 5 μL injection; autosampler 15 °C. The gradient was 0–0.25 min, 50% B; 0.25–1.25 min, ramp to 100% B; 1.25–3.0 min, hold 100% B; 3.1 min, return to 50% B; 3.1–4.0 min, re-equilibrate.

Mass spectrometry used positive electrospray ionization (ESI+) with nitrogen (GS1 = 35 psi; GS2 = 70 psi; curtain gas = 40 psi; CAD = 10; source 500 °C; 5,500 V). Multiple-reaction monitoring transitions were: ascomycin 809.3 → 756.4 (CE 29 V, EP 10 V, CXP 13 V); tacrolimus 821.3 → 768.4 (CE 25 V, EP 10 V, CXP 11 V); rapamycin 931.6 → 864.5 (CE 35 V, EP 10 V, CXP 15 V).

Calibration curves were linear (R^2^ > 0.999) over 1–40 ng/mL for TAC and 0.5–25 ng/mL for RAPA; LLOQ were 2 ng/mL (TAC) and 0.5 ng/mL (RAPA). Intra- and inter-assay variability were <10%. Samples were analyzed in triplicate, and peaks were integrated in Analyst 3.0. Concentrations were reported as ng/mL blood (or ng/mg tissue, normalized to wet weight).

### Depot retrieval

Healthy male Lewis rats received a single subcutaneous PRECISE injection (0.25 mL; 7 mg TAC, 6 mg RAPA, n = 3). After a week, the animals underwent surgical depot retrieval on POD 7.

Rats were anesthetized with isoflurane (3–4% induction, 1.5–2% maintenance) and given pre-emptive analgesia (buprenorphine SR, 1.0 mg/kg SC). The injection site was shaved, prepped with alternating povidone-iodine and 70% ethanol, and draped. A 1–1.5 cm skin incision was made directly over the palpable depot. The depot, which had solidified by phase inversion, was exposed by blunt and limited sharp dissection through subcutaneous tissue. The intact implant was freed from the fibrous pocket using fine scissors, avoiding fragmentation, and removed en bloc. Skin was closed with 4–0 nylon interrupted sutures and covered with topical antibiotic ointment. Rats recovered in a warmed cage and received postoperative analgesia (buprenorphine 0.05 mg/kg SC every 12 h for 48 h). No antibiotics were used routinely.

### Rat hindlimb allotransplantation

Animals were anesthetized with inhalation anesthesia (SomnoSuite^®^, Kent Scientific, CT, USA), and a sterile technique was used for all the surgical procedures. Both donor and host rats were simultaneously prepared for the limb transplantation by two microsurgeons. In the donor operation, the skin was incised proximal to the mid-thigh area, the iliac vessels and nerve were dissected, and the individual muscle groups were divided proximally. The femur was divided at the mid-shaft. The limb was perfused with cold preservative solution and stored on ice until transplantation. In the host operation, the bone was fixed using an intramedullary pin. Donor iliac vessels and nerves were anastomosed end-to-end to recipient femoral vessels with 10–0 nylon under microscopy, and the sciatic and femoral nerves were coapted with an epineural technique. The muscles were reapproximated using 5–0 Vicryl sutures, and the skin was closed using absorbable 5–0 Monocryl suture (Ethicon, Inc., Cincinnati, OH). Buprenorphine (sustained-release, 0.05 mg/kg SC) was administered pre-operatively. Animals were followed for up to 300 days or until Banff grade 3 rejection. Animals were allocated to three cohorts: (i) untreated controls (n = 12), (ii) systemic TAC comparator (IP TAC 1 mg/kg/day, n = 6), and (iii) PRECISE cohort (n = 12). In the PRECISE cohort, a 0.25 mL subcutaneous depot of TAC/RAPA hydrogel (7 mg/mL TAC; 6 mg/mL RAPA; 1.75 mg and 1.50 mg per animal; 5.83 mg/kg and 5.00 mg/kg) was placed at surgery, split between the dorsal and ventral aspects of the graft. Identical 0.25 mL maintenance depots were administered at monthly intervals from POD 30 through POD 300, totaling 11 administrations. The systemic TAC comparator received daily IP injections (1 mg/kg/day) beginning on POD 0.

Animals were monitored daily for signs of limb rejection. Important clinical signs included edema, erythema, escharification, and necrosis. Peeling skin on minimal pressure was considered confirmation of frank rejection. All animals were weighed daily for 2 months and weekly thereafter. Animals were euthanized upon Banff grade 3 rejection or at study endpoint.

### Porcine gracilis free flap allotransplantation

Domestic crossbred Landrace × Yorkshire × Duroc (L × Y × D) pigs (6–8 weeks old; 15–20 kg) underwent reciprocal gracilis myocutaneous free-flap exchanges (≈5 × 10 × 4 cm) transplanted heterotopically to the recipient neck. Donor–recipient pairs were fully mismatched at the SLA loci. Under general anesthesia, the flap pedicle was anastomosed end-to-end to the carotid artery and jugular vein using 8–0 nylon. Three cohorts were studied: (i) no immunosuppression; (ii) systemic steroid induction alone (intraoperative solumedrol 500 mg IV followed by 100 mg per day on POD 1–4, then discontinued); and (iii) PRECISE plus systemic steroid induction (same steroid regimen). Local analgesia (0.25% bupivacaine) was provided at incision sites. In the PRECISE cohort, PRECISE hydrogel depots (drug-loaded or unloaded) were injected at multiple subcutaneous sites across the flap at the time of transplantation. Local analgesia (0.25% bupivacaine) was provided at incision sites. For postoperative assessments, animals were sedated with Telazol (5–8 mg/kg IM) on POD 1, 2, 7, 14, and weekly thereafter; euthanasia was performed under isoflurane overdose at study endpoints or upon Banff grade 3 rejection. Grafts were assessed clinically for perfusion, color, temperature, edema, and skin integrity at the above time points.

### Swine leukocyte antigen (SLA) genotyping and donor–recipient matching

All animals underwent SLA genotyping prior to selection as previously described (PMID 19392823 and 20121817) with modifications made to the typing primer panels to broaden allele coverage and enhance typing resolution in outbred farm pigs. The class I genes SLA-1, SLA-2, and SLA-3, and the class II genes DRB1, DQB1, and DQA were analyzed. High-resolution, allelic SLA typing was inferred based on previous literature and reports [PMID 15713212, 16305679, 19317739]. Donor-recipient pairs were blood-type compatible, but either partially or fully mismatched for their SLA class I and class II antigens.

### Histopathology

Skin punch biopsies were obtained at defined time points and at necropsy (2 mm in rats; 6 mm in pigs). Specimens were fixed in 10% neutral-buffered formalin, paraffin-embedded, sectioned (5 μm), and stained with hematoxylin–eosin (H&E) for routine histopathologic evaluation. Additional samples were snap-frozen in OCT for IHC.

Rejection severity was graded according to the modified Banff VCA criteria(29), defined as follows: Grade 0, no or rare infiltrates; Grade I, mild, focal infiltrates without epidermal necrosis; Grade II, moderate, multifocal infiltrates with early epidermal necrosis; Grade III, severe, diffuse infiltration with epidermal necrosis; and Grade IV, necrotizing acute rejection. Chronic rejection was assessed by quantifying intimal hyperplasia (IH) and luminal occlusion on H&E- and elastin-stained vessels using ImageJ software.

### Mixed lymphocyte reaction (MLR) assay in rats

PBMCs from PRECISE–treated long-term surviving Lewis recipients and naïve Lewis rats were isolated by Ficoll-Hypaque density gradient and used as responders. Responders were labeled with CFSE (Thermo Fisher Scientific) at 5 μM for 10 minutes at room temperature, quenched by adding five volumes of complete medium, washed twice, and resuspended in complete RPMI-1640. Donor-strain BN splenocytes were prepared by mechanical dissociation, subjected to ACK red blood cell lysis, washed, and irradiated at 3,000 rad to serve as stimulators. Responder and stimulator cells were combined at a 2:1 ratio in 96-well U-bottom plates in RPMI-1640 media with 10% fetal bovine serum, 1% penicillin–streptomycin, 2 mM L-glutamine, and 50 μM 2-mercaptoethanol. Cultures were incubated for 7 days at 37 °C in 5% CO_2_. At the end of the culture period, proliferation was quantified by CFSE dilution on a BD LSR Fortessa cytometer and analyzed in FlowJo.

### Flow cytometry for immune cell phenotyping

For immunophenotyping, PBMCs were isolated from postoperative samples using Ficoll-Hypaque (density 1.077 g/L; TBD Sciences). PBMCs were stained with fluorescently labeled antibodies targeting cell surface markers CD4, CD25, and the intracellular transcription factor FoxP3 (BD Biosciences, San Diego, CA). All stained cells were subsequently analyzed using a BD LSR Fortessa cytometer, and the data was analyzed with FlowJo software (Tree Star, Ashland, OR).

## Supplementary Material

1
