# Supplementary material for: A Precisely Controlled Long-Acting Immunosuppression Platform Enables Prolonged Survival of Vascularized Composite Allografts
Source: bioRxiv. 2025 Nov 10:2025.11.07.687240. Preprint. [Version 1] doi: 10.1101/2025.11.07.687240 (PMC12642550; doi:10.1101/2025.11.07.687240)
Supplement: 1 [file NIHPP2025.11.07.687240V1-supplement-1.pdf]

bioRxiv preprint doi: https://doi.org/10.1101/2025.11.07.687240; this version posted November 10, 2025. The copyright holder for this preprint (which was not certified by peer review) is the author/funder, who has granted bioRxiv a license to display the preprint in perpetuity. It is made available under aCC-BY 4.0 International license.

Supplementary Figures

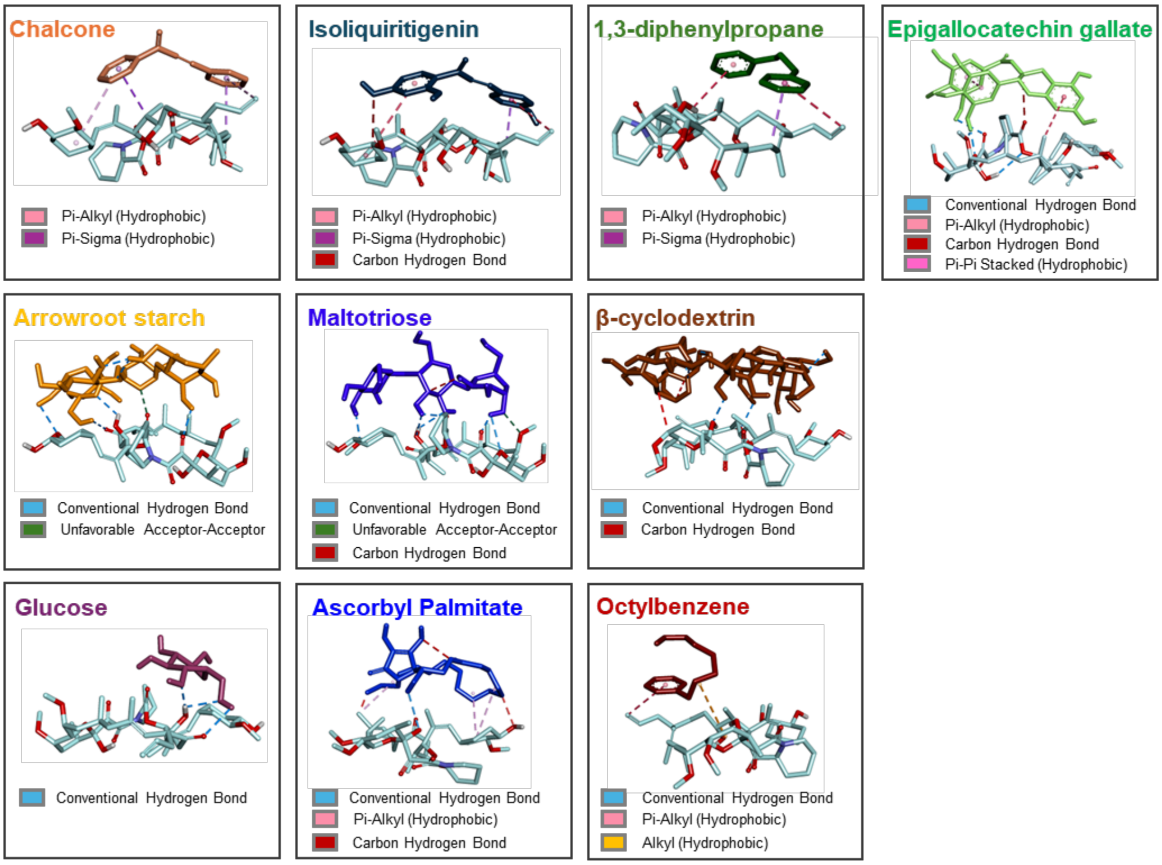

Figure S1. Representative *in silico* docking poses (AutoDock Vina) of TAC with GRAS-listed DBAs. Compounds were classified into three structural groups—aromatic polyphenols, carbohydrates, and long-chain hydrophobes—for experimental validation.

bioRxiv preprint doi: https://doi.org/10.1101/2025.11.07.687240; this version posted November 10, 2025. The copyright holder for this preprint (which was not certified by peer review) is the author/funder, who has granted bioRxiv a license to display the preprint in perpetuity. It is made available under aCC-BY 4.0 International license.

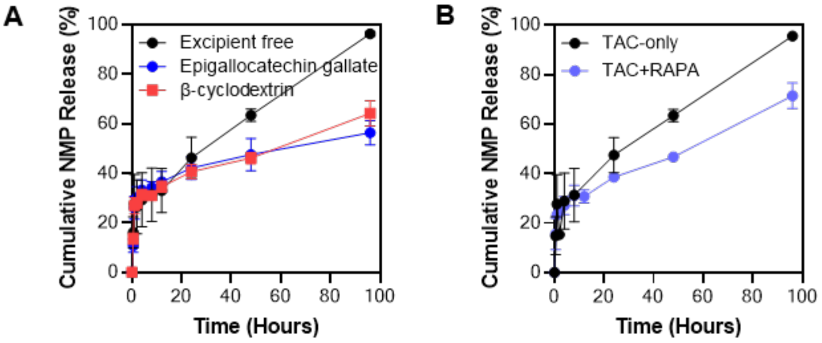

Figure S2. Drug–excipient modulation of NMP efflux during depot formation. (A) Cumulative NMP release from PRECISE depots with or without DBAs (EGCG and β-cyclodextrin). Both excipients slowed solvent diffusion relative to excipient-free PRECISE, reducing early solvent efflux and thus limiting burst release. **(B)** Cumulative NMP release from TAC-only and TAC+RAPA PRECISE depots showing that RAPA co-loading attenuates NMP efflux, suggesting that TAC-RAPA interactions modulate solvent dynamics during phase inversion.

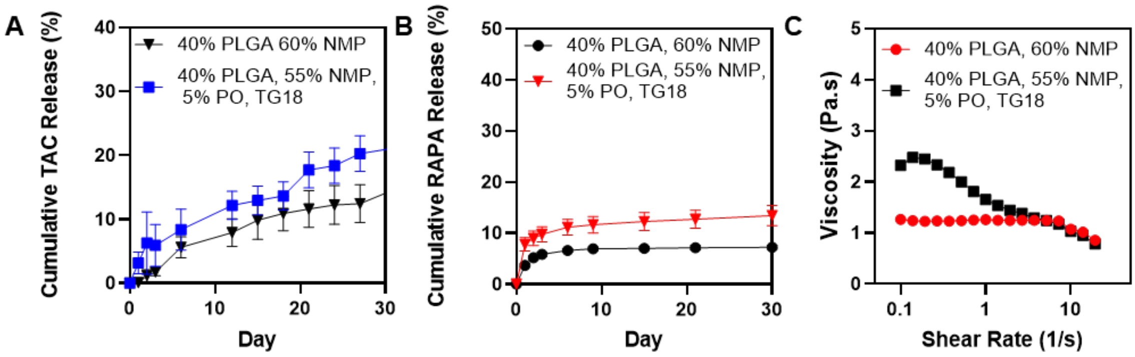

Figure S3. Shear-thinning formulation for on-bed dosing preserves PRECISE release kinetics. (A) Rotational rheometry of the modified PRECISE (5 wt% NMP replaced with palm oil; TAC pre-encapsulated in lyophilized TG-18 and dispersed in the PLGA/NMP phase) exhibits pronounced shear-thinning relative to the original PLGA/NMP PRECISE, enabling easy injection and rapid viscosity recovery for stable wound-bed depot formation. **(B–C)** In vitro cumulative release of **(B)** TAC and **(C)** RAPA from the modified PRECISE overlaps with the original PLGA/NMP PRECISE without palm oil/TG-18, indicating that the additives do not increase burst or alter long-term kinetics. Conditions: mean ± SEM; n as indicated; 37 °C; PBS with 20% (v/v) methanol.

bioRxiv preprint doi: https://doi.org/10.1101/2025.11.07.687240; this version posted November 10, 2025. The copyright holder for this preprint (which was not certified by peer review) is the author/funder, who has granted bioRxiv a license to display the preprint in perpetuity. It is made available under aCC-BY 4.0 International license.

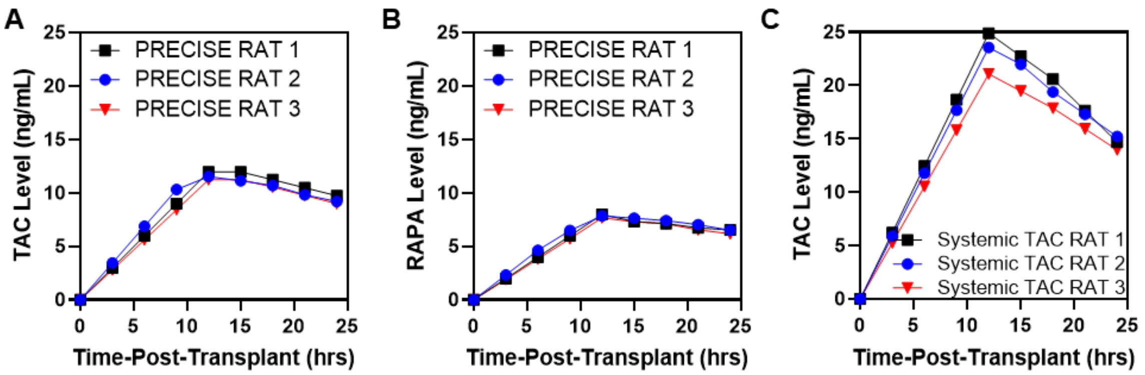

Figure S4. Early-phase (0–24 h) pharmacokinetics in rats. Early-phase (0–24 h) pharmacokinetic profiles of **(A)** TAC and **(B)** RAPA after a single subcutaneous PRECISE injection, compared with **(C)** daily intraperitoneal TAC. Concentrations were sampled over 24 h and used for $AUC_{0\text{-}24\ h}$ calculations shown in Figure 4D.

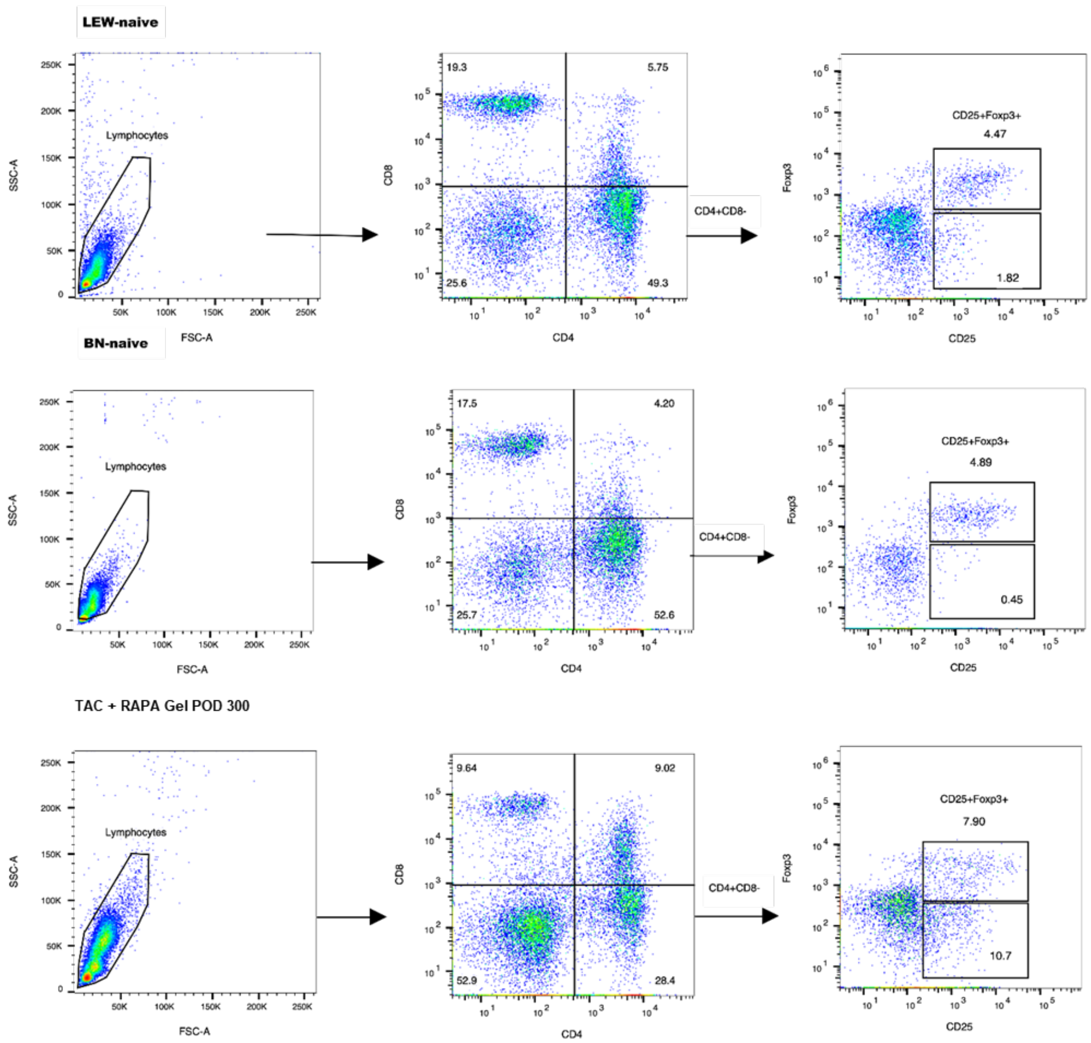

Figure S5. Flow cytometry gating and longitudinal regulatory T-cell analysis in rats. Sequential gating strategy for identification of $CD4^{+}CD25^{+}Foxp3^{+}$ Tregs from peripheral blood mononuclear cells

bioRxiv preprint doi: https://doi.org/10.1101/2025.11.07.687240; this version posted November 10, 2025. The copyright holder for this preprint (which was not certified by peer review) is the author/funder, who has granted bioRxiv a license to display the preprint in perpetuity. It is made available under aCC-BY 4.0 International license.

(PBMCs). Representative dot plots from naïve and PRECISE-treated rats at POD 30, 160, and 300 showing expansion of the Treg population.

		DONOR						
	Animal ID	P21063 A 32.1/62.12a	P21064 A 32.1/32.15b	P21065 A 31.13a/32.1	P21066 Non-A 40.11/58.WF1	P21067 Non-A 34.23/40.11	P21068 Non-A 45.11/59.27	P21069 A 32.1/58.WF1
RECIPIENT	P21063 A 32.1/62.12a	—	1 Class II haplotype mismatch	1 haplotype mismatch	2 haplotype mismatch	2 haplotype mismatch	2 haplotype mismatch	2 haplotype mismatch
	P21064 A 32.1/32.15b	1 haplotype mismatch	—	1 haplotype mismatch	2 haplotype mismatch	2 haplotype mismatch	2 haplotype mismatch	2 haplotype mismatch
	P21065 A 31.13a/32.1	1 haplotype mismatch	1 Class II haplotype mismatch	—	2 haplotype mismatch	2 haplotype mismatch	2 haplotype mismatch	2 haplotype mismatch
	P21066 Non-A 40.11/58.WF1	Blood type incompatible	Blood type incompatible	Blood type incompatible	—	1 haplotype mismatch	2 Class I and II haplotype mismatch	Blood type incompatible
	P21067 Non-A 34.23/40.11	Blood type incompatible	Blood type incompatible	Blood type incompatible	1 haplotype mismatch	—	2 Class I and II haplotype mismatch	Blood type incompatible
	P21068 Non-A 45.11/59.27	Blood type incompatible	Blood type incompatible	Blood type incompatible	2 Class I and II haplotype mismatch	2 Class I and II haplotype mismatch	—	Blood type incompatible
	P21069 A 32.1/58.WF1	1 haplotype mismatch	1 Class II haplotype mismatch	1 haplotype mismatch	1 haplotype mismatch	2 haplotype mismatch	2 haplotype mismatch	—

Figure S6. Donor–recipient pairs were selected to maximize immunological disparity using a compatibility matrix based on swine leukocyte antigen (SLA) haplotypes. SLA Class I (SLA-1, SLA-2, SLA-3) and Class II (DRB1, DQB1) typing was performed using low-resolution PCR and high-resolution sequence-based genotyping. Haplotypes were assigned according to IPD-MHC SLA database nomenclature. SLA-matched pairs and ABO-incompatible pairs were excluded to avoid immunological permissiveness or hyperacute rejection, respectively. Final pairs reflected full Class I and II mismatches. Two reciprocal donor–recipient combinations were selected to assess directionality under maximal allogeneic pressure.

bioRxiv preprint doi: https://doi.org/10.1101/2025.11.07.687240; this version posted November 10, 2025. The copyright holder for this preprint (which was not certified by peer review) is the author/funder, who has granted bioRxiv a license to display the preprint in perpetuity. It is made available under aCC-BY 4.0 International license.

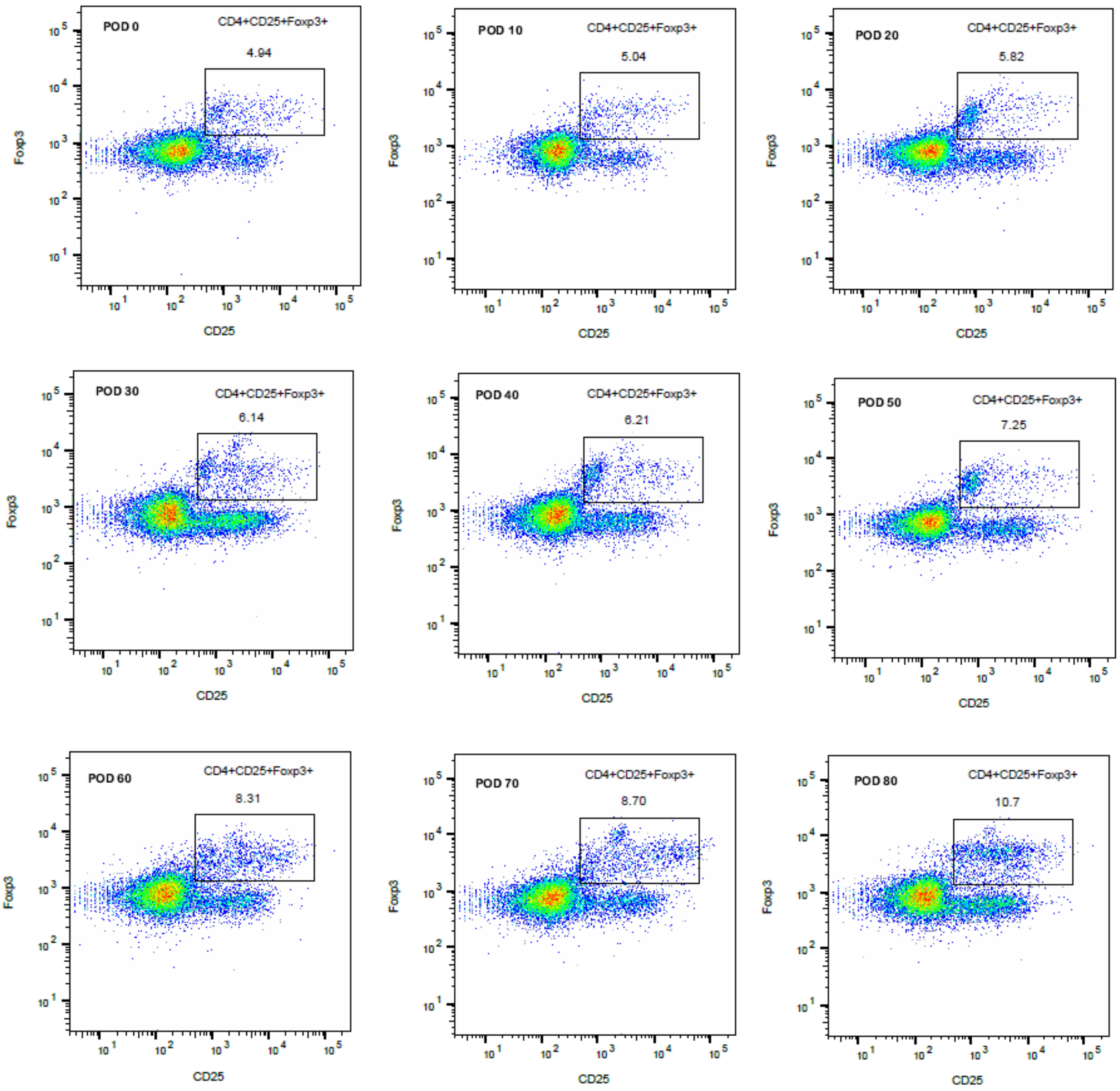

Figure S7. Flow cytometry of peripheral blood mononuclear cells (PBMCs) in the PRECISE + steroid cohort showed a progressive increase in CD4⁺CD25⁺Foxp3⁺ Tregs from ~5% at baseline (POD 0) to ~11% by POD 80.

Table

Table S1. HPLC conditions for in-vitro tacrolimus quantification.

Time (min)	Methanol (%)	Water (%)	Flow rate (mL/min)
0	70	30	
3	70	30	
15	90	10	1
16	90	10	
19	70	30	
